# What Is the Role of Resilience and Coping Strategies on the Mental Health of the General Population during the COVID-19 Pandemic? Results from the Italian Multicentric COMET Study

**DOI:** 10.3390/brainsci11091231

**Published:** 2021-09-17

**Authors:** Gaia Sampogna, Valeria Del Vecchio, Vincenzo Giallonardo, Mario Luciano, Umberto Albert, Claudia Carmassi, Giuseppe Carrà, Francesca Cirulli, Bernardo Dell’Osso, Giulia Menculini, Mariagiulia Nanni, Maurizio Pompili, Gabriele Sani, Umberto Volpe, Valeria Bianchini, Andrea Fiorillo

**Affiliations:** 1 Department of Psychiatry, University of Campania Luigi Vanvitelli, Largo Madonna delle Grazie, 80138 Naples, Italy; valeria.delvecchio78@gmail.com (V.D.V.); enzogiallo86@gmail.com (V.G.); mario.luciano@unicampania.it (M.L.); andrea.fiorillo@unicampania.it (A.F.); 2 Department of Medicine, Surgery and Health Sciences, University of Trieste, 34128 Trieste, Italy; ualbert@units.it; 3 Department of Mental Health, Psychiatric Clinic, Azienda Sanitaria Universitaria Giuliano-Isontina—ASUGI, 34128 Trieste, Italy; 4 Department of Clinical and Experimental Medicine, University of Pisa, 56126 Pisa, Italy; ccarmassi@gmail.com; 5 Department of Medicine and Surgery, University of Milan Bicocca, 20126 Milano, Italy; giuseppe.carra@unimib.it; 6 Center for Behavioral Sciences and Mental Health, National Institute of Health, 00161 Rome, Italy; francesca.cirulli@iss.it; 7 Department of Mental Health, Department of Biomedical and Clinical Sciences “Luigi Sacco”, University of Milan, 20157 Milan, Italy; bernardo.dellosso@unimi.it; 8 Department of Health Sciences, Aldo Ravelli Center for Neurotechnology and Brain Therapeutic, University of Milan, 20142 Milan, Italy; 9 Department of Psychiatry, University of Perugia, 06123 Perugia, Italy; giuliamenculini@gmail.com; 10 Department of Biomedical and Specialty Surgical Sciences, Institute of Psychiatry, University of Ferrara, 44121 Ferrara, Italy; nnnmgl@unife.it; 11 Department of Neurosciences, Mental Health and Sensory Organs, Faculty of Medicine and Psychology, Sapienza University of Rome, 00185 Roma, Italy; maurizio.pompili@uniroma1.it; 12 Department of Neuroscience, Section of Psychiatry, University Cattolica del Sacro Cuore, 00168 Rome, Italy; gabriele.sani@unicatt.it; 13 Department of Psychiatry, Fondazione Policlinico Agostino Gemelli IRCCS, 00168 Rome, Italy; 14 Clinical Psychiatry Unit, Department of Clinical Neurosciences, Università Politecnica delle Marche, 60121 Ancona, Italy; u.volpe@staff.univpm.it; 15 Department of Life, Health and Environmental Sciences, Psychiatric Unit: Trattamenti Riabilitativi Psicosociali, Interventi Precoci, TRIP, Psychosocial Rehabilitation Treatment, Early Interventions University Unit, University of L’Aquila, 67100 L’Aquila, Italy; valeria.bianchini@univaq.it

**Keywords:** resilience, coping strategies, trauma, pandemic, mental health

## Abstract

The effects of the COVID-19 pandemic on mental health are now well documented, however, few studies have been focused on the role of coping strategies and resilience in counterbalancing these detrimental effects. Data are derived from the COvid Mental hEalth Trial (COMET), a national multicentric trial carried out in the Italian general population. The final sample consisted of 20,720 participants, 53.1% (*n* = 11,000) of the sample reported low levels of resilience. Adaptive coping strategies and resilience levels did not have any significant protective impact on the levels of depressive, anxiety, and stress symptoms. Only self-distraction was a risk factor for poor mental health (Beta Coefficient, B = 0.1, 95% Confidence Interval, CI: 0.003 to 0.267 for stress symptoms; B = 0.2; 95% CI: 0.077 to 0.324 for anxiety symptoms and B = 0.2, 95% CI: 0.105 to 0.382 for depressive symptoms). High levels of resilience were predicted by adaptive coping strategies, such as acceptance (B = 1.8, CI 95% = 1.4–2.7). Exposure to the different weeks of lockdown, being infected by COVID-19, and being a healthcare professional did not influence the levels of resilience. Our findings should be carefully considered, since the low levels of resilience may represent the missing link between the pandemic and the current increase in mental health problems.

## 1. Introduction

The COVID-19 pandemic is an unprecedented life-threatening event that is affecting the mental and physical health and well-being of the general population worldwide [1,2,3]. It has been considered as a new traumatic experience, which is completely different from all other natural or man-made disasters [4,5,6]. The traumatic role of the COVID-19 pandemic is due to its direct and indirect threats to important life resources of the general population, such as safety, health, income, work, housing, and social support [7,8,9,10].

The effects of the COVID-19 pandemic on mental health have been well documented. Trials carried out in the last year have identified differential effects in samples including the general population, health professionals, those affected by COVID-19, people with disabilities, or affected by chronic physical and mental health conditions. In particular, the psychiatric and psychological consequences of the pandemic on the general population mainly include high levels of distress, insomnia, depressive and anxiety symptoms [11,12,13,14,15,16,17,18,19]. Health professionals are at high risk of developing burn-out and insomnia [20,21,22,23,24]. In disabled people and in those with pre-existing mental health problems, an increased risk of treatment interruption has been found, associated with relapses or symptoms worsening [25,26,27,28,29]. Finally, people affected by COVID-19 have experienced high levels of trauma-related disorders, cognitive deficits, and depression [30,31,32,33,34]. Specific risk factors identified for the development of these mental health disturbances include female gender, having previous mental health or physical disorders, loneliness, time spent on the Internet, and unemployment [11,25].

Although the general population as a whole is exposed to the same traumatic event, the perception of the pandemic is highly variable, being mediated by individual psychological and social strengths, including coping strategies and resilience styles.

Coping strategies are defined as the skills needed to manage and adjust to stressful situations, representing an essential element for the adaptation process to stressful and traumatic situations as well as for the recovery process of patients with severe mental disorders [35]. Lazarus and Folkman [36] identified problem-oriented and emotion-focused coping strategies. The former includes practical strategies, such as seeking information and positive communication, in order to deal with the stressful situation and are associated with a better long-term outcome for patients and relatives; the latter are psychologically driven, such as venting or avoidance, and are associated with a worse outcome [22,37].

Resilience is defined as a positive growth or adaptation following periods of homeostatic disruption [38]. This positive adaptation in response to extreme adversities was originally thought to characterize extraordinary individuals; more recently, it has been shown that resilience is relatively common also among children and adolescents exposed to adversity and trauma [39,40].

Several studies have been carried out so far in order to identify the prevalence of personal or social factors protecting people from developing mental disorders during the pandemic [41,42,43,44,45,46]. In particular, studies have been focused on the perceived levels of stress, resilience, and coping strategies related to COVID-19 in the general population [47,48], in older adults [45,49], in pregnant women [50,51,52,53], in college students [54], in children and adolescents [55,56], and in mental health professionals [57,58].

Based on the largest Italian study evaluating the effects of the COVID-19 pandemic on the mental health of the general population [11], in this paper we aimed to: (1) describe the levels of coping strategies and resilience in the Italian general population during the first wave of the pandemic; (2) evaluate the protective role of coping strategies and resilience on the levels of depressive, anxiety and stress symptoms at DASS-21 scale [59]; (3) assess the relationship between the levels of resilience and respondents’ psychiatric symptoms, socio-demographic characteristics and coping strategies.

## 2. Materials and Methods

The COvid Mental hEalth Trial (COMET) is a national trial coordinated by the University of Campania “Luigi Vanvitelli” (Naples) in collaboration with nine university sites: Università Politecnica delle Marche (Ancona), University of Ferrara, University of Milan Bicocca, University of Milan “Statale”, University of Perugia, University of Pisa, Sapienza University of Rome, “Catholic” University of Rome, University of Trieste. The Center for Behavioral Sciences and Mental Health of the National Institute of Health in Rome has been involved in the study by supporting the dissemination and implementation of the project according to the clinical guidelines produced by the National Institute of Health for managing the effects of the COVID-19 pandemic.

The COMET was conceived as a cross-sectional observational design using a snowball sampling method for the recruitment of the Italian general population. The full study protocol is available elsewhere [60].

The main outcome measure of the study is the DASS-21, evaluating the general distress on a tripartite model of psychopathology [59]. It consists of 21 items grouped in three subscales: depression, anxiety, and stress. The depression scale assesses dysphoria, hopelessness, devaluation of life, self-deprecation, lack of interest/involvement, anhedonia and inertia. The anxiety scale assesses autonomic arousal, skeletal muscle effects, situational anxiety, and subjective experience of anxious affect. The stress scale evaluates the levels of chronic nonspecific arousal. It assesses difficulty relaxing, nervous arousal, and being easily upset/agitated, irritable/over-reactive and impatient. Each item is rated on a 4-level Likert scale, from 0 (never) to 3 (almost always). The total score is calculated by adding together the response value of each item, with higher scores indicating more severe levels of depressive, anxiety, and stress symptoms. The score at the DASS–depression subscale (e.g., “I felt that I had nothing to look forward to”) is divided into normal (0–9), mild (10–12), moderate (13–20), severe (21–27), and extremely severe depression (28–42). The score at the DASS–anxiety subscale (e.g., “I was worried about situations in which I might panic and make a fool of myself”) is divided into normal (0–6), mild (7–9), moderate (10–14), severe (15–19), and extremely severe anxiety (20–42). The score at the DASS–stress subscale (e.g., “I tended to over-react to situations”) is divided into normal (0–10), mild (11–18), moderate (19–26), severe (27–34), and extremely severe stress (35–42).

The levels of resilience have been evaluated by the Connor Davidson Resilience Scale (CD-RISC), which includes 10 items rated on a 6-level Likert scale. Higher values indicate higher levels of resilience [61]. As reported by Campbell-Sills et al. [62], the levels of resilience can be subdivided into quartiles, from lowest to highest quartiles: 0–29 indicate low resilience, 30–32 median resilience, 33–36 moderate resilience, and 37–40 high resilience.

Coping strategies have been evaluated using the Brief-COPE, consisting of 28 items grouped in 14 subscales [63]. Each item is rated on a 4-level Likert scale from 0 = “I have not been doing this at all” to 3 = “I have been doing this a lot”. Coping strategies are grouped into maladaptive strategies, including denial, venting, behavioral disengagement, self-blame, self-distraction, and substance abuse, and adaptive coping strategies, which include emotional support, use of information, positive reframing, planning, and acceptance. Two other subscales include religion and humor.

Respondents’ socio-demographic (e.g., gender, age, geographical region, working and housing condition, etc.) and clinical information (e.g., having a previous physical or mental disorder, using illicit drugs or medications, etc.) have been collected through ad-hoc schedules. Other validated and reliable questionnaires included in the study are: the General Health Questionnaire—12 items version (GHQ) [64]; the Obsessive-Compulsive Inventory—Revised version (OCI-R) [65]; the Insomnia Severity Index (ISI) [66]; the Suicidal Ideation Attributes Scale (SIDAS) [67]; the Severity of Acute Stress Symptoms Adult Scale (SASS) [68]; the Impact of Event Scale—short version (IES) [69]; the UCLA loneliness scale—short version [70]; the short form of Post-Traumatic Growth Inventory (PTGI) [71]; the Multidimensional Scale of Perceived Social Support (MSPPS) [72] and (only for healthcare professionals) the Maslach Burnout Inventory (MBI) [73].

### Statistical Analysis

Descriptive statistics were performed in order to describe the socio-demographic and clinical characteristics of the sample according to the different quartiles of resilience. Chi-square with multiple comparisons and ANOVA with Bonferroni corrections were performed for evaluating differences according to quartiles of resilience in the type of coping strategies adopted, as well as in the levels of depressive, anxiety, and stress symptoms, insomnia, post-traumatic growth, and perceived loneliness.

Multivariate linear regression models were implemented for testing the role of resilience levels and of coping strategies as predictors of the levels of depressive, anxiety, and stress symptoms (primary outcome evaluated with DASS-21). The models were adjusted for the rate of new COVID-19 cases and of COVID-19-related mortality during the study period, as well as for several socio-demographic characteristics, such as gender, age, occupational status, having a physical comorbid condition, having a pre-existing mental disorder, employment status, unemployment due to the pandemic and levels of perceived loneliness. Furthermore, in order to evaluate the impact of the duration of lockdown and of other related containment measures on the primary outcomes, the categorical variable “Week” was also entered in the regression models. Interaction terms have also been created for testing the impact of coping strategies/resilience with main socio-demographic and clinical variables.

In order to identify possible predictors of the levels of resilience, a multivariate linear regression model, weighted for the propensity score, was performed, including as independent variables: adaptive and maladaptive coping strategies, having been infected by COVID-19, having a pre-existing mental disorder, and being a healthcare professional. Furthermore, in order to evaluate the impact of the duration of lockdown and of other related containment measures on the primary outcomes, the categorical variable “Week” was also entered in the regression models. The models were adjusted for the rate of new COVID-19 cases and of COVID-19-related mortality during the study period, as well as for several socio-demographic characteristics, such as gender, age, occupational status, having a physical comorbid condition, hours spent on Internet, levels of perceived loneliness, health status, number of co-habitants, satisfaction with one’s own life, and with housing conditions. All variables have been managed as reported in detail elsewhere [25].

Missing data have been handled using the multiple imputation approach. Statistical analyses were performed using the Statistical Package for Social Sciences (SPSS), version 17.0, and STATA, version 15. For all analyses, the level of statistical significance was set at *p* < 0.05.

## 3. Results

### 3.1. Global Sample

The final sample consisted of 20,720 participants, 71% female (*n* = 14,720), with a mean age of 40.4 (14.3) years; half of the respondents were in a stable relationship and were living with a partner (Table 1).

**Figure 1:**
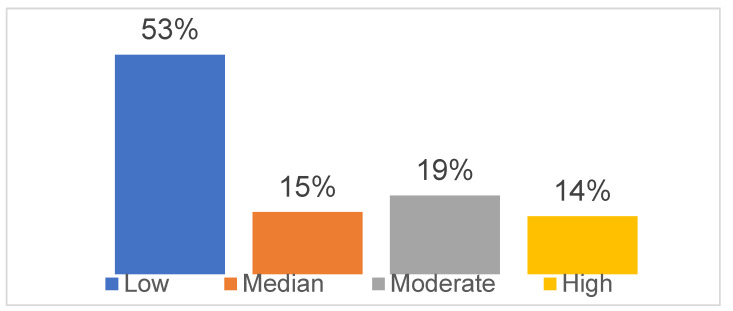
Levels of resilience in the global sample (*n* = 20,270).

**Figure 2:**
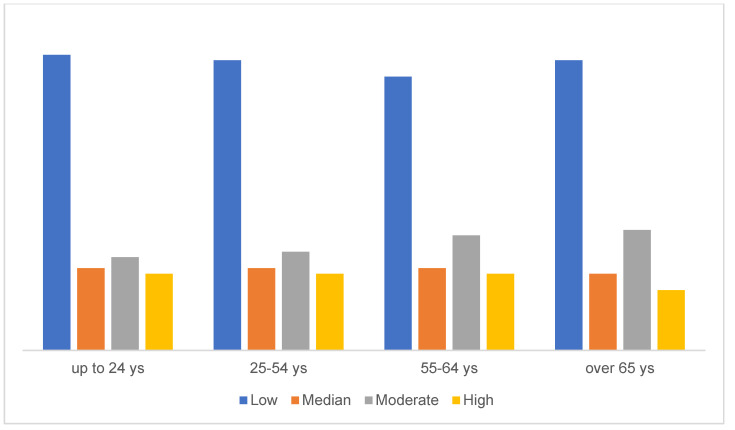
Levels of resilience according to the age group (*n* = 20,270).

More than half of the sample (*n* = 11,000; 53.1%) reported low levels of resilience, which were not associated with age (r = 0.008, *p* = 0.235) or gender (χ^2^ = 0.860, *p* = 0.301) (Figure 1 and Figure 2); however, higher levels of resilience were found in people with a higher level of education (r = 0.017, *p* < 0.005). The levels of resilience did not differ among the general population, patients with pre-existing mental disorders and those infected by COVID-19 (Table 2).

### 3.2. Differences in the Sample According to the Levels of Resilience

Low levels of resilience were associated with more severe insomnia (Insomnia Severity Index, ISI mean score: 6.9 ± 5.3), depressive symptoms (DASS-Depression mean score: 12.6 ± 7.5), and higher levels of perceived loneliness (UCLA mean score: 12.1 ± 3.3) (Table 3).

People with low versus high levels of resilience did not differ in intrusive, avoidance, and hyperarousal symptoms at Impact of Event Scale (IES), suicidal ideation, personal possibilities, and sense of closeness with others at Post-Traumatic Growth Inventory (PTGI). On the other hand, highly resilient people reported a significantly higher level of appreciation for life (Table 3).

People with low levels of resilience rarely used positive coping strategies. For example, only 22.5% of those with low levels of resilience used “planning” as a strategy as compared to 54.2% of people identified as having high resilience (*p* < 0.0001). “Acceptance” was used in 28.1% of cases among those with low levels of resilience in contrast with 60.7% in people with high levels of resilience (*p* < 0.0001). On the other hand, maladaptive coping strategies, such as behavioral disengagement was adopted by 63.8% of people in the low resilience group compared to 48.3% in the high resilience group (*p* < 0.0001).

### 3.3. Impact of Coping Strategies and Resilience on Mental Health Status

According to the multivariate regression models, weighted for the propensity score, adaptive coping strategies did not have any influence on the levels of depressive and anxiety symptoms, even controlling for the impact of age, gender, presence of pre-existing mental/physical conditions, as well as the levels of loneliness. Only practical support is associated with lower levels of stress symptoms, with a Beta coefficient (B) of −0.186 (95% CI: −0.371 to −0.001) (Table 3). Among the maladaptive coping strategies, only self-distraction was a risk factor for poor mental health, with a Beta coefficient of 0.162 (95% CI: 0.038 to 0.286) for anxiety symptoms, and B = 0.182 (95% CI: 0.044 to 0.321) for depressive symptoms (Table 3). The levels of resilience did not have any influence on stress symptoms (B = −0.001, *p* < 0.984), depressive symptoms (B = −0.008; *p* < 0.230) or anxiety symptoms (B = −0.010, *p* < 0.075) (Table 4).

Being female, older, having a pre-existing mental or physical condition were significantly associated with higher levels of depressive, anxiety and stress symptoms.

Interaction terms (i.e., COPE emotional disengagement * pre-existing mental disorder) have been created and included in the models, but no significant effects were identified (Supplementary Materials Table S1).

### 3.4. Impact of Coping Strategies on Resilience Levels

According to the multivariate regression model, weighted for the propensity score, high levels of resilience were predicted by the presence of adaptive coping strategies, such as acceptance (B = 1.8, CI 95% = 1.4–2.7), planning (B = 2.1, CI 95% = 1.7–2.5), and positive reframing (B = 1.3, CI 95% = 0.9–1.6). Interestingly, the levels of resilience were reduced by other adaptive coping strategies, such as the search for information (B = −1.1, 95% CI =−1.5 to −0.6) and emotional support (B = −1.2, 95% CI = −1.6 to −0.7).

On the contrary, low levels of resilience were predicted by maladaptive coping strategies, including self-blame (B = −0.6, CI 95% = −0.9 to −0.3), emotional disengagement (B = −1.0, CI 95% = −1.4 to −0.6), venting (B = −0.3, CI 95% = −0.7 to −0.3) and self-distraction (B = −0.7, CI 95% = −1 to −0.4), and by higher levels of perceived loneliness (B = −0.3, 95% CI = −0.39 to −0.24).

The exposure to the different weeks of lockdown, being infected by COVID-19, and being a healthcare professional did not influence the levels of resilience, even after controlling for gender, age, the presence of physical or psychiatric comorbidities.

The levels of depressive, anxiety, or stress symptoms, insomnia, post-traumatic symptoms, and suicidal ideation did not have any influence on the levels of resilience (Table 3).

Finally, high levels of post-traumatic growth, such as identifying new possibilities (B = 1.5, 95% CI = 1.2–1.8) and improving own personal strengths (B = 1.5, 95% CI = 1.3–1.8) were significant protective factors for high levels of resilience (Table 5).

## 4. Discussion

The study herein describes resilience levels in a large sample of the Italian general population during the initial phase of the pandemic. This data provides valuable insight into how the pandemic affected a Westernized country. Our findings should be carefully considered in order to develop ad hoc supportive and preventive psychosocial interventions for limiting the long-term detrimental effects of the pandemic on mental health.

The most striking finding is the presence of low levels of resilience, as reported by the majority of the sample. Since the pandemic is leading to increased levels of mental health concerns among the general population [11,25], this finding suggests that there may be a link between psychiatric symptoms and low levels of resilience [39,74].

We did not find any difference in the levels of resilience according to gender and age, although previous studies found that women are more resilient than men, and that older people are more resilient than younger ones [75,76,77,78]. This finding may suggest that this pandemic has the same effects on the whole population, regardless of gender and age. We did find a relationship between resilience and education, confirming that the development of resilience is influenced by education and by culture. However, the mean years of education of our sample were quite high and cannot be considered fully representative of the Italian general population.

During crises, people adopt different coping strategies [79], as confirmed in our sample from the general population adopting a variety of coping strategies to overcome the situation. In particular, people using self-distraction or behavioral disengagement more frequently were less likely to have high levels of resilience. This is in line with data from another survey carried out in Australia, showing that maladaptive coping strategies are associated with high levels of anxiety and distress symptoms [80].

The main hypothesis of our study, i.e., the protective role of adaptive coping strategies and resilience in the presence of depressive, anxiety, and stress symptoms, was not confirmed by our results. In fact, the most relevant factors associated with poor mental health were female gender, become unemployed due to the pandemic, the duration of lockdown, being infected by COVID-19, and having a pre-existing mental health problem [25]. These findings should be interpreted with caution as the results were likely influenced by the severe impact of the pandemic and the rapid containment measures that were implemented which is historically unprecedented.

One of the main aims of the study was to identify the predictors of the levels of resilience in the general population. People using positive coping strategies, such as planning, acceptance and reframing, have higher levels of resilience. This is in line with a study carried out on health care professionals, which showed that having positive attitudes in the workplace is associated with low levels of distress [57], greater psychological wellbeing, and better quality of life [81,82].

An interesting finding is related to the use of searching for information as an adaptive coping strategy, which appears to be a negative predictor of resilience. This finding highlights the negative role held by media during the pandemic, with sensationalistic (and sometimes biased) news rapidly spread through social media, and the tendency of the general population to look for information more frequently than in the past [83]. For this tendency, the WHO has coined the term “infodemic”, suggesting its maladaptive role as a coping strategy [84,85]. Furthermore, there is the need for a better collaboration between health professionals and journalists in order to provide the general population with correct, balanced, and unambiguous information on the pandemic [86].

The presence of maladaptive coping strategies is a significant risk factor for low levels of resilience. This finding confirms data from previous epidemics, where people adopting avoidance or denial more frequently reported an increase in the levels of distress and a reduction in resilience [87,88]. The predictive role of coping strategies on the levels of resilience should be considered in psychiatric routine clinical practice, since the adoption of adaptive coping strategies can be improved through ad hoc psychoeducational interventions [89,90].

We also found that the levels of resilience were not influenced by the duration of lockdown, having been infected by the COVID-19 and being a healthcare professional. An earlier study carried out in Italy on healthcare professionals found that resilience was lower in healthcare professionals than in the general population after the first COVID-19 wave [91], but the small sample size may have influenced the predictive power of those findings. In our sample, we did not find any association between the role of healthcare professionals and the levels of resilience.

It is interesting that the levels of resilience were not influenced by geographical factors, infection, and mortality rates by COVID-19, nor by the duration of exposure to lockdown. In previous analyses based on the COMET study, we found that the duration of lockdown was a significant risk factor for the levels of depressive, anxiety and stress symptoms in the general population [25]. However, while the duration of lockdown and related containment measures is an evolving process, resilience is more stable as it is related to the individual’s cognitive style, personality traits, and temperament [92,93,94,95,96]. These aspects, which have not been considered in the current paper, will be analyzed in forthcoming analyses in order to verify the role of personality traits as possible mediators of coping strategies and resilience.

The present study has some limitations which are hereby acknowledged. First, the snowball sampling methodology could have led to a selection bias, with only those interested in the psychological consequences of the pandemic willing to participate [97]. Second, the cross-sectional design of the survey prevents us to delineate any causal relationship between the selected variables. Finally, several variables which could have an impact on coping strategies and on resilience levels, such as family functioning, family communication styles, and acceptance of restrictive measures related to the pandemic, have not been collected in our study.

## 5. Conclusions

The assessment of the levels of resilience and of coping strategies adopted by the general population in the context of different types of adverse situations is of great importance for the development of ad hoc supportive and preventive psychosocial interventions. It is well established that the pandemic will have longstanding, and far-reaching, consequences on global mental health and wellness. Therefore, the identification of those factors which may explain the causality between the pandemic and its impact on mental health will be crucial from a public health perspective. The presence of low levels of resilience in the general population may be the missing link between the pandemic and increasing concerns surrounding mental health.

## Figures and Tables

**Table 1:** Socio-demographic and clinical characteristics of the sample (*n* = 20,720).

Age, years, mean ± SD	40.4 ± 14.3
Age groups, % (*n*)	
18–24 years old	15.2 (3151)
25–55 years old	65.2 (13,514)
55–64 years old	14.0 (2904)
over 65 years old	5.6 (1151)
Gender, F, % (*n*)	71 (14,720)
Living with partner, yes, % (*n*)	52.2 (10,808)
University degree, yes, % (*n*)	62 (12,844)
Employed, yes, % (*n*)	70 (14,518)
Lost job due to the pandemic, yes, % (*n*)	6.3 (1302)
Are you practicing smart working, yes, % (*n*)	34.2 (7089)
Spending more time on Internet, yes, % (*n*)	80.1 (16,598)
Any comorbid physical condition(s), yes, % (*n*)	14.5 (3012)
Any mental health problem(s), yes, % (*n*)	5.5 (1133)
Have you been infected by COVID-19, yes, % (*n*)	1.4 (296)
Have you been isolated due to COVID-19 infection, yes, % (*n*)	1.5 (316)
Have you been in contact with someone affected by COVID-19, % (*n*)	4.2 (866)

**Table 2:** Differences among groups in the levels of resilience.

	Levels of Resilience M (SD)	*p* Value
General population	28.3 (10.4)	0.368
People with pre-existing mental disorders	28.1 (10.7)	0.288
People with COVID-19 infection	28.4 (10.4)	0.849
People with pre-existing physical disorders	28.1 (10.4)	0.223

**Table 3:** Differences in clinical characteristics according to the levels of resilience.

	Levels of Resilience				
	Low	Median	Moderate	High	*p* Value
Insomnia severity index, M (SD)	6.9 (5.3) ^a^	6.7 (5.1)	6.6 (5.1)	6.0 (5.2) ^a^	0.002
DASS Anxiety subscale, M (SD)	7.6 (6.8)	7.3 (6.8)	7.3 (6.7)	7.6 (6.9)	0.070
DASS Depression subscale, M (SD)	12.6 (7.5) ^a^	12.1 (7.6)	12.4 (7.3)	12.0 (7.3) ^a^	0.001
DASS Stress subscale, M (SD)	16.4 (7.1)	16.5 (7.2)	16.2 (6.9)	16.3 (7.1)	0.286
GHQ total score, M (SD)	17.6 (3.2)	17.4 (3.0)	17.4 (3.1)	17.4 (3.2)	0.386
IES Avoidance, M (SD)	2.3 (1.9)	2.3 (1.9)	2.4 (2.0)	2.4 (1.9)	0.701
IES Hyperarousal, M (SD)	2.5 (1.9)	2.5 (1.8)	2.6 (1.9)	2.6 (1.9)	0.201
IES Intrusiveness, M (SD)	2.1 (1.9)	2.1 (1.9)	2.1 (2.0)	2.1 (1.9)	0.662
PTGI—Appreciation for life, M (SD)	2.1 (1.3)	2.4 (1.3) ^a^	2.5 (1.4)	2.5 (1.4) ^a^	0.0001
PTGI—New possibilities, M (SD)	1.4 (1.1) ^a^	1.9 (1.1)	2.0 (1.2)	2.2 (1.3) ^a^	0.0001
PTGI—Personal strength, M (SD)	1.6 (1.3) ^a^	2.2 (1.3)	2.5 (1.4)	2.7 (1.5) ^a^	0.0001
PTGI—Relating to others, M (SD)	1.6 (1.3) ^a^	1.9 (1.3)	2.1 (1.4)	2.2 (1.4) ^a^	0.0001
PTGI—Spiritual help, M (SD)	0.9 (1.1)	1.2 (1.1)	1.3 (1.2)	1.3 (1.1)	0.0001
SASS global score, M (SD)	0.8 (0.7)	0.7 (0.7)	0.8 (0.7)	0.8 (0.7)	0.389
SIDAS global score, M (SD)	4.9 (6.8)	4.6 (6.6)	4.5 (5.8)	4.8 (6.7)	0.490
Support—Significant others, M (SD)	21.1 (7.1) ^a^	22.4 (6.3)	23.2 (6.1)	23.4 (6.1) ^a^	0.0001
Support—Friends, M (SD)	18.6 (6.8) ^a^	20.6 (6.0)	21.2 (5.9)	21.8 (5.8) ^a^	0.0001
Support—Family, M (SD)	19.7 (6.9) ^a^	21.5 (6.2)	21.9 (6.2)	22.4 (6.2) ^a^	0.0001
UCLA global score, M (SD)	2.0 (0.5) ^a^	1.9 (0.5)	1.8 (0.5)	1.7 (0.5) ^a^	0.0001

DASS, Depression, anxiety, and Stress scale; IES, Impact of Event Scale; GHQ, General Health Questionnaire; PTGI, Post-Traumatic Growth Inventory; SASS, Severity of Acute Stress Symptoms; UCLA, UCLA loneliness scale; M, mean; SD, standard deviation; ^a^ pairwise comparison, *p* < 0.005.

**Table 4:** Impact of coping strategies and resilience on mental health status.

	DASS Stress				DASS Anxiety				DASS Depression			
	B	Sign.	95% Confidence Interval		B	Sign.	95% Confidence Interval		B	Sign.	95% Confidence Interval	
			Lower Bound	Upper Bound			Lower Bound	Upper Bound			Lower Bound	Upper Bound
Intercept	18.292	0.000	16.740	19.844	8.871	0.000	7.427	10.316	15.352	0.000	13.733	16.971
COPE Active coping	−0.002	0.977	−0.159	0.154	0.030	0.683	−0.115	0.176	−0.056	0.500	−0.219	0.107
COPE Denial	0.037	0.644	−0.119	0.193	0.076	0.304	−00.069	0.221	0.008	0.920	−0.154	0.171
COPE Substance abuse	0.048	0.608	−0.135	0.231	0.097	0.266	−0.074	0.267	0.061	0.534	−0.130	0.251
COPE Emotional support	0.094	0.311	−0.088	0.277	−0.055	0.530	−0.225	0.116	0.047	0.626	−0.143	0.238
COPE Practical support	**−0.186**	**0.049**	**−0.371**	**−0.001**	0.002	0.985	−0.171	0.174	−0.082	0.403	−0.275	0.111
COPE Emotional disengagement	0.134	0.120	−0.035	0.302	0.070	0.381	−0.087	0.227	0.103	0.252	−0.073	0.278
COPE Venting	0.095	0.233	−0.061	0.250	0.087	0.239	−0.058	0.232	0.088	0.287	−0.074	0.250
COPE Reframing	−0.095	0.203	−0.241	0.051	0.126	0.069	−0.010	0.262	−0.055	0.481	−0.207	0.098
COPE Planning	**0.216**	**0.014**	**0.044**	**0.387**	0.118	0.149	−0.042	0.278	0.112	0.221	−0.067	0.291
COPE Humor	0.084	0.243	−0.057	0.225	0.125	0.063	−0.007	0.256	−0.019	0.801	−0.166	0.128
COPE Acceptance	−0.077	0.341	−0.235	0.081	−0.139	0.065	−0.286	0.009	−0.002	0.978	−0.168	0.163
COPE Religion	0.031	0.625	−0.094	0.157	0.001	0.988	−0.116	0.118	−0.020	0.770	−0.151	0.112
COPE Self-blame	−0.079	0.281	−0.221	0.064	0.013	0.847	−0.120	0.146	−0.027	0.727	−0.175	0.122
COPE Self-distraction	0.101	0.138	−0.032	0.234	**0.162**	**0.010**	**0.038**	**0.286**	**0.182**	**0.010**	**0.044**	**0.321**
Resilience levels	0.000	0.984	−0.012	0.012	−0.010	0.075	−0.021	0.001	−0.008	0.230	−0.020	0.005
Gender, ref. male	**2.061**	**0.000**	**1.849**	**2.272**	**2.086**	**0.000**	**1.889**	**2.283**	**1.693**	**0.000**	**1.471**	**1.914**
Age	**0.056**	**0.000**	**−0.063**	**−0.049**	**−0.062**	**0.000**	**−0.069**	**−0.055**	**−0.052**	**0.000**	**−0.059**	**−0.044**
Quarantine, yes	−0.238	0.546	−1.010	0.534	−0.226	0.538	−0.945	0.493	**−1.072**	**0.009**	**−1.878**	**−0.267**
Being infected by COVID, yes	0.262	0.438	−0.400	0.924	**1.552**	**0.000**	**0.935**	**2.169**	**1.452**	**0.000**	**0.761**	**2.143**
Healthcare professional, yes	0.217	0.561	−0.514	0.947	0.051	0.883	−0.629	0.731	−0.180	0.644	−0.942	0.582
Mental disorder, yes	0.672	0.093	−0.112	1.456	**4.534**	**0.000**	**3.804**	**5.265**	**3.871**	**0.000**	**3.053**	**4.689**
Pre-existing physical condition, yes	**0.787**	**0.000**	**0.504**	**1.707**	**1.490**	**0.000**	**1.227**	**1.754**	**0.995**	**0.000**	**0.699**	**1.269**
Employed, yes	−0.269	**0.018**	−0.491	−0.047	0.611	0.000	0.404	0.818	1.324	0.000	1.092	1.556
Lost job, yes	0.696	**0.001**	0.299	1.093	**1.219**	**0.000**	**0.850**	**1.589**	**2.231**	**0.000**	**1.817**	**2.645**
Time to exposure, ref. week March 30–April 8												
Week April 15–April 9	**1.642**	**00.000**	**0.866**	**2.418**	**2.444**	**00.000**	**1.721**	**3.166**	**1.645**	**00.000**	**0.836**	**2.455**
Week April 16–April 22	**1.520**	**00.000**	**0.937**	**2.102**	**1.842**	**00.000**	**1.299**	**2.384**	**1.481**	**00.000**	**0.874**	**2.089**
Week April 23–April 29	**0.935**	**00.000**	**0.430**	**1.439**	**1.009**	**00.000**	**0.539**	**1.478**	**0.810**	**0.003**	**0.283**	**1.336**
Week April 30–May 4	**0.349**	**0.041**	**0.015**	**0.684**	**0.341**	**0.032**	**0.029**	**0.653**	**0.387**	**0.030**	**0.038**	**0.736**
PTGI—Relating to others	0.080	0.123	−0.022	0.181	0.085	0.078	−0.009	0.180	0.050	0.353	−0.056	0.156
PTGI—New possibilities	−0.005	0.933	−0.113	0.104	−0.099	0.054	−0.200	0.002	−0.058	0.316	−0.171	0.055
PTGI—Personal strength	−0.068	0.304	−0.197	0.062	−0.127	0.039	−0.248	−0.007	−0.065	0.349	−0.200	0.071
PTGI—Spiritual help	−0.014	0.768	−0.107	0.079	0.024	0.594	−0.063	0.110	0.023	0.643	−0.074	0.120
PTGI—Appreciation life	0.005	0.938	−0.123	0.133	**0.198**	**0.001**	**0.079**	**0.318**	**0.216**	**0.002**	**0.083**	**0.350**
Support from others	0.011	0.238	−0.007	0.030	0.011	0.221	−0.007	0.028	−0.010	0.322	−0.029	0.010
Support from friends	−0.011	0.258	−0.030	0.008	−0.017	0.058	−0.034	0.001	**−0.022**	**0.029**	**−0.041**	**−0.002**
Support from family	−0.009	0.318	−0.027	0.009	−0.008	0.358	−0.025	0.009	−0.007	0.480	−0.026	0.012
UCLA Loneliness	0.011	0.518	−0.023	0.045	0.010	0.523	−0.021	0.042	**0.045**	**0.013**	**0.009**	**0.080**
Cases COVID-19	00.000	0.101	−00.000	0.001	00.000	0.108	−6.967	00.000	00.000	0.132	−2.086	00.000
Death COVID-19	00.000	0.621	−0.002	0.001	0.002	0.109	−00.000	0.003	0.001	0.385	−0.001	0.002
Model statistics	20.300 (39), *p* < 0.001 0.035				48.315 (39), *p* < 00.000 0.082				34.732 (39), *p* < 0.001 0.060			
F (df), *p*-value Adjusted R^2^												

PTGI—Post Traumatic Growth Inventory; DASS, Depression, Anxiety, Stress scale; COPE, Brief—COPE. Significant *p* values are highlighted in bold characters.

**Table 5:** Predictors of resilience levels.

	B	*p*-Value	95% Confidence Interval	
			Lower Bound	Upper Bound
Intercept	13.221	0.000	9.786	16.656
Time to exposure, ref. week March 30–April 8				
Week April 15–April 9	1.197	0.177	−0.540	2.934
Week April 16–April 22	0.614	0.358	−0.695	1.924
Week April 23–April 29	0.941	0.105	−0.196	2.078
Week April 30–May 4	0.236	0.534	−0.508	0.980
Age, ref. >34 ys				
Age 18–25 ys	0.204	0.421	−0.293	0.701
Age 25–28 ys	0.072	0.840	−0.629	0.773
Gender female, ref. Male	−0.171	0.500	−0.669	0.326
Having a pre-existing mental health problem	0.141	0.775	−0.827	1.110
Being health care professional	−0.384	0.241	−1.026	0.257
Having been infected by COVID	−0.031	0.952	−1.031	0.970
Being in one of the most affected Italian regions	−0.478	0.046	−0.948	−0.008
Adaptive coping strategies				
Acceptance	**1.826**	**0.000**	**1.474**	**2.179**
Emotional support	**−1.171**	**0.000**	**−1.580**	**−0.762**
Planning	**2.091**	**0.000**	**1.711**	**2.470**
Active	**0.622**	**0.000**	**0.273**	**0.970**
Positive reframing	**1.258**	**0.000**	**0.934**	**1.582**
Use of information	**−1.088**	**0.000**	**−1.499**	**−0.676**
*Maladaptive coping strategies*				
Self-blame	**−0.591**	**0.000**	**−0.912**	**−0.270**
Denial	−0.046	0.802	−0.402	0.311
Substance abuse	0.333	0.120	−0.087	0.752
Emotional disengagement	**−1.030**	**0.000**	**−1.406**	**−0.654**
Venting	−0.332	0.067	−0.689	0.024
Self-distraction	**−0.706**	**0.000**	**−1.006**	**−0.406**
*Other coping strategies*				
Humor	**2.284**	**00.000**	**1.970**	**2.597**
Religion	**−0.816**	**0.000**	**−1.097**	**−0.535**
PTGI—Appreciation life	**−0.449**	**0.000**	**−0.658**	**−0.239**
PTGI—New possibilities	**1.464**	**0.000**	**1.173**	**1.755**
PTGI—Personal strength	**1.540**	**0.000**	**1.297**	**1.784**
PTGI—Relating to others	**−0.264**	**0.023**	**−0.491**	**−0.036**
PTGI—Spiritual help	−0.032	0.829	−0.323	0.259
DASS_Stress	−0.014	0.426	−0.049	0.021
DASS Depression	0.020	0.256	−0.015	0.056
DASS Anxiety	−0.031	0.126	−0.070	0.009
IES global score	0.030	0.152	−0.011	0.070
ISI global score	−0.024	0.360	−0.074	0.027
SASS global score	0.252	0.280	−0.205	0.710
GHQ global score	0.013	0.704	−0.055	0.081
SIDAS global score	0.005	0.761	−0.029	0.040
OCI global score	−0.022	0.225	−0.056	0.013
UCLA global score	**−0.321**	**0.000**	**−0.398**	**−0.243**
Support from others	0.018	0.407	−0.025	0.061
Support from friends	**0.211**	**0.000**	**0.169**	**0.253**
Support from family	**0.091**	**0.000**	**0.051**	**0.132**
Cases COVID-19	0.010	0.285	0.000	0.001
Death COVID-19	−9.224	0.956	−0.003	0.003
Model statistics				
F (df), *p*-value Adjusted R^2^	81.704 (41), *p* < 0.000 0.388			

DASS, Depression, Anxiety and Stress scale; IES, Impact of Event Scale; GHQ, General Health Questionnaire; PTGI, Post-Traumatic Growth Inventory; SASS, Severity of Acute Stress Symptoms; UCLA, UCLA loneliness scale; OCI—obsessive-compulsive inventory; ISI—Insomnia severity index; B, Beta coefficient. Significant *p*-values are highlighted in bold characters; Italics and underline indicate the different subscales.

## Data Availability

The data presented in this study are available on request from the corresponding author.

## Supplementary Materials

Supplementary materials are available online at https://www.mdpi.com/article/10.3390/brainsci11091231/s1, Table S1: Impact of coping strategies and resilience on mental health status, adjusted for interaction terms.
