# Supplementary material for: What Is the Role of Resilience and Coping Strategies on the Mental Health of the General Population during the COVID-19 Pandemic? Results from the Italian Multicentric COMET Study
Source: Brain Sci. 2021 Sep 17;11(9):1231. doi: 10.3390/brainsci11091231 (PMC8466446; doi:10.3390/brainsci11091231)
Supplement: Supplementary file 1 [file brainsci-11-01231-s001.zip › brainsci-1363420-supplementary.pdf]

Supplementary Material. Table S1. Impact of coping strategies and resilience on mental health status. adjusted for interaction terms

	DASS stress				DASS Anxiety				DASS Depression			
	B	Sign.	95% Confidence Interval		B	Sign.	95% Confidence Interval		B	Sign.	95% Confidence Interval	
			Lower bound	Upper bound			Lower bound	Upper bound			Lower bound	Upper bound
**Intercept**	18..927	..000	16..524	21..330	10.573	.000	8.609	12.538	16.328	.000	13.878	18.779
COPE Active coping	-..002	..975	-..159	..154	.032	.668	-.114	.178	-.055	.508	-.219	.108
COPE Denial	..035	..662	-..121	..191	.078	.293	-.067	.223	.010	.900	-.152	.173
COPE Substance abuse	..048	..604	-..134	..231	.100	.248	-.070	.271	.063	.516	-.128	.254
COPE Emotional support	..094	..312	-..089	..277	-.059	.500	-.229	.112	.044	.653	-.147	.235
COPE Practical support	.-.203	..519	-..818	..413	.005	.957	-.167	.177	-.080	.417	-.273	.113
COPE Emotional disengagement	..134	..119	-..034	..303	.073	.362	-.084	.230	.104	.246	-.072	.280
COPE Venting	..094	..234	-..061	..250	..086	..245	-..059	..231	..088	..288	-..074	..250
COPE Reframing	-..095	..204	-..241	..052	..122	.079	-.014	.258	-.058	.453	-.211	.094
COPE Planning	..015	..962	-..603	..633	.122	.136	-.038	.282	.115	.210	-.064	.294
COPE Humor	..083	..248	-..058	..224	.126	.060	-.005	.257	-.018	.807	-.166	.129
COPE Acceptance	-..076	..350	-..234	..083	-.140	.063	-.287	.008	-.002	.978	-.168	.163
COPE Religion	..031	..627	-..095	..157	.000	.999	-.117	.117	-.020	.762	-.151	.111
COPE Self-blame	-..078	..287	-..220	..065	.014	.834	-.119	.147	-.025	.740	-.174	.124
COPE Self-distraction	..102	..132	-..031	..235	-.481	.068	-.997	.035	-.188	.602	-.892	.517
Resilience levels	..000	..985	-..012	..012	-.010	.077	-.021	.001	-.008	.235	-.020	.005
Gender, ref. male	**2..061**	**..000**	**1..849**	**2..272**	**2.086**	**.000**	**1.889**	**2.283**	**1.693**	**.000**	**1.471**	**1.914**
Age	**-..056**	**..000**	**-..063**	**-.049**	**-.062**	**.000**	**-.069**	**-.055**	**-.052**	**.000**	**-.059**	**-.044**
Quarantine, yes	-..236	..549	-1..008	.536	-.220	.548	-.940	.499	-1.067	.009	-1.873	-.262
Being infected by COVID, yes	..261	..441	-..402	..923	1.475	.044	.038	2.911	1.448	.000	.757	2.139
Healthcare professional, yes	..215	..565	-..516	..945	.048	.891	-.633	.728	-.184	.637	-.946	.579
Mental disorder, yes	..670	..094	-..115	1.454	5.067	.000	3.626	6.509	4.527	.000	2.912	6.142
Pre-existing physical condition, yes	**..787**	**..000**	**..504**	**1.707**	**1.490**	**.000**	**1.227**	**1.754**	**.995**	**.000**	**.699**	**1.269**
Employed, yes	-..144	..756	-1..055	.766	.409	.227	-.255	1.072	1.227	.001	.485	1.970
Lost job, yes	-..987	..255	-2..685	.712	-2.323	.000	-3.565	-1.081	-2.811	.000	-4.203	-1.419
Time to exposure, ref. week March 30 – April 8												
Week April 15 – April 9	**1..645**	**..000**	**..869**	**2.421**	**2.435**	**.000**	**1.712**	**3.157**	**1.639**	**.000**	**.829**	**2.448**
Week April 16- April 22	**1..522**	**..000**	**..939**	**2.104**	**1.846**	**.000**	**1.304**	**2.388**	**1.484**	**.000**	**.876**	**2.092**
Week April 23 – April 29	**..936**	**..000**	**..431**	**1.440**	**1.005**	**.000**	**.535**	**1.474**	**.806**	**.003**	**.280**	**1.332**
Week April 30 – May 4	**..351**	**..040**	**..017**	**.686**	**.343**	**.031**	**.031**	**.654**	**.388**	**.029**	**.039**	**.737**
PTGI - Relating to others	..080	..124	-..022	.181	.084	.080	-.010	.179	.050	.358	-.056	.156
PTGI - New possibilities	-..005	..934	-..113	.104	-.098	.057	-.199	.003	-.057	.321	-.170	.056
PTGI - Personal strenght	-..069	..299	-..198	.061	-.127	.039	-.248	-.007	-.065	.349	-.200	.071
PTGI - Spiritual help	-..014	..764	-..107	.079	.022	.620	-.065	.108	.022	.660	-.075	.119
PTGI - Appreciation life	..006	..930	-..122	.134	.201	.001	.082	.320	.218	.001	.084	.352
Support from others	..011	..245	-..008	.030	.011	.220	-.006	.028	-.010	.321	-.029	.010
Support from friends	-..011	..260	-..030	.008	-.017	.060	-.034	.001	-.022	.030	-.041	-.002
Support from family	-..009	..321	-..027	.009	-.008	.360	-.025	.009	-.007	.484	-.026	.012
UCLA Loneliness	..011	..517	-..023	.045	.010	.542	-.022	.041	.050	.354	-.056	.156
Cases COVID	..000	..101	-..000	.001	.000	.109	6.4815	.000	.000	.335	1.705	.000
Death COVID	..000	..624	-..002	.001	.002	.108	-.000	.003	.001	.373	-.001	.002
**Interaction terms**												
Pre-existing physical condition * COPE practical support	..007	..969	-..343	.357	-	-	-	-	-	-	-	-
Employment * COPE practical support	-..081	..550	-..346	.184	-	-	-	-	-	-	-	-
Lost job * COPE practical support	..039	..886	-..488	.566	-	-	-	-	-	-	-	-
Lost job * COPE planning	..066	..809	-..466	.597	-	-	-	-	-	-	-	-
Employment * COPE planning	..024	..863	-.253	.301	-	-	-	-	-	-	-	-
Pre-existing physical condition* COPE planning	..154	..400	-..205	.513	-	-	-	-	-	-	-	-
Employment * COPE Emotional disengagement	-	-	-	-	.077	.520	-.158	.313	-	-	-	-
Lost job * COPE Emotional disengagement	-	-	-	-	.412	.068	-.031	.854	.216	.394	-.280	.712
Pre-existing mental disorder * COPE Emotional disengagement	-	-	-	-	-.197	.402	-.660	.265	-.244	.357	-.762	.274
Pre-existing physical condition * COPE Emotional disengagement	-	-	-	-	.285	.068	-.021	.591	.225	.197	-.117	.568
Being infected by COVID * COPE Emotional disengagement	-	-	-	-	.027	.912	-.453	.507	-	-	-	-
Employment * COPE Emotional disengagement	-	-	-	-	-	-	-	-	.037	.781	-.226	.301

COPE Emotional disengagement * UCLA loneliness	-	-	-	-	-	-	-	-	-.002	.913	-.039	.035
**Model statistics**												
F (df). p value	17.618 (45). p<.001				43.015 (44). p<.000				30.865 (44). p <.001			
Adjusted R^2	.035				.082				.060			

DASS, Depression, Anxiety and Stress scale; PTGI, Post-Traumatic Growth Inventory; UCLA, Ucla loneliness scale; COPE, Brief – COPE scale; B, Beta coefficient
Significant p-values are highlighted in bold characters
